# Pharmacology: Clinical and Experimental

**Published:** 1914-10

**Authors:** 


PHARMACOLOGY: CLINICAL AND EXPERIMENTAL. Dr. Hans Meyer of Vienna and Dr. R. Gottlieb of Heidelberg. Authorized translation into English by John Taylor Holsey, M. D., Professor of Pharmacology, Therapeutics and Clinical Medicine, Tulane University. From the press of J. B. Lippincott Co., Philadelphia. Price, $6.00.

From the standpoint of the student of pharmacology this is an excellent book, well written, and covering all the phases of the physiological action of drugs in disease. For the general practitioner who seeks higher knowledge along the lines of physiology many vistas of deep thought are opened up. He is led to see the great "Why" of many things that are only hazy recollections of what his professor of physiology endeavored to fix on the tablets of his memory.

For this work the authors have gone deeply into very interesting subject matter,—the behavior of living organisms under chemically altered conditions of life; in other words, what happens when drugs are administered.

Scientific drug therapy, explaining the ways and means by which various pathological conditions may be influenced by drugs, must go hand in hand with general pathology, and it is here that the work is particularly good and worth far more than its price.

The translation into English is excellent. There is little of the stretching of meaning of words to convey the idea expressed in the original, and Dr. Holsey's notes on the text are all valuable.

As for the printing, type and binding, they are but further illustrations of the excellence which the house of Lippincott has attained in these arts. The book is well worth the price of $6.00 which has been set upon it. Its index and references to literature on the many subjects discussed are excellent.

